# Evaluation of Preoperative Capnographic Parameters in Children with Suspected Foreign Body Aspiration

**DOI:** 10.3390/children12121710

**Published:** 2025-12-18

**Authors:** Murat Kuru, Tamer Altinok, Resul Yılmaz

**Affiliations:** 1 Thoracic Surgery Department, Necmettin Erbakan University School of Medicine, 42080 Konya, Turkey; 2 Anesthesiology and Reanimation Department, Necmettin Erbakan University School of Medicine, 42080 Konya, Turkey

**Keywords:** foreign bodies, bronchoscopy, child, blood gas analysis, oxygen saturation

## Abstract

**Objective:** To evaluate the clinical and biochemical differences between pediatric patients with suspected foreign body aspiration (FBA) who had foreign bodies detected on bronchoscopy and those who did not. **Methods:** Patients undergoing bronchoscopy for suspected FBA were retrospectively divided into two groups: Group 1 (n = 59), with confirmed foreign body; Group 2 (n = 50), without foreign body. Age, blood gas parameters (pCO_2_, pO_2_, SpO_2_), and type and localization of foreign bodies were recorded and statistically compared. **Results:** The mean age was significantly lower in Group 1 (24.63 ± 12.32 months) than in Group 2 (37.12 ± 32.98 months; *p* = 0.014). Group 1 had significantly higher pCO_2_ levels (41.24 ± 13.37 mmHg vs. 31.53 ± 6.44 mmHg; *p* < 0.001) and lower pO_2_ levels (45.78 ± 12.18 mmHg vs. 53.98 ± 13.24 mmHg; *p* = 0.001). Oxygen saturation values showed no significant difference between groups (*p* = 0.19). Among confirmed cases, foreign bodies were located in the right bronchial system (56%), left bronchial system (41%), and trachea (3.4%). **Conclusions:** Children diagnosed with FBA were younger and exhibited greater abnormalities in blood gas parameters compared to those without FBA. While bronchoscopy remains essential for the definitive diagnosis and treatment of suspected FBA, our findings suggest that these results may play a significant role in reducing unnecessary bronchoscopies.

## 1. Introduction

Foreign body aspiration (FBA) is defined as the entry of a foreign material into the tracheobronchial system during respiration. FBA is particularly common in the pediatric population between 1 and 3 years of age and represents a potentially life-threatening condition. While it may present acutely with clinical manifestations such as cough, dyspnea, stridor, and asphyxia, chronic cases may be complicated by recurrent pneumonia or air trapping [1]. Approximately two-thirds of deaths related to FBA occur immediately at home following the event; however, in patients who reach the hospital, mortality rates are reported between 0 and 1.5% [2]. Multiple studies have emphasized that aspirated foreign bodies are most commonly lodged in the right main bronchus, with organic materials such as peanuts and almonds being predominant, and that urgent bronchoscopic removal is usually required [3].

Traditional diagnostic tools for FBA include physical examination, radiological imaging, and bronchoscopy, while the use of capnography and arterial/venous blood gas measurements has been very rarely detailed in the literature. Capnography, which is the monitoring of CO_2_ concentration in expired breath, and capnometry, which is the measurement of CO_2_ concentration, are non-invasive methods increasingly used to assess ventilation in critically ill patients. In particular, systematic investigations focusing on partial pressure of carbon dioxide (pCO_2_) values are extremely limited. Case reports have suggested that hypercapnia may develop in patients with ventilation insufficiency, reflecting airway obstruction as part of the pathophysiology of FBA [4,5]. Especially in “ball-valve” type obstructions, distal air trapping and impaired gas exchange are likely to occur [6]. Thus, quantitative data derived from pCO_2_ measurements could potentially contribute to the diagnostic process of FBA. However, a systematic analysis statistically correlating pCO_2_ levels with the diagnosis and ventilatory impact of FBA is still lacking.

Preoperative oxygen saturation (SpO_2_) and the partial pressure of oxygen (pO_2_) values, although not sufficiently evaluated in the literature, have been considered potentially useful in determining the severity of suspected FBA and the necessity for bronchoscopy in anecdotal and small-scale studies. In a retrospective study, Zhang et al. reported that operative duration, foreign body size, and presence of pneumonia during bronchoscopy increased the risk of hypoxemia, thereby highlighting the importance of preoperative oxygenation parameters [7].

The aim of the present study was to analyze the role of preoperatively recorded pCO_2_, pO_2_, and SpO_2_ values in detecting the presence of tracheobronchial foreign bodies in patients presenting with suspected FBA. By doing so, we aimed to reduce unnecessary use of bronchoscopy, particularly in high-risk patients with comorbidities and increased morbidity and mortality.

## 2. Methods

**Figure 1:**
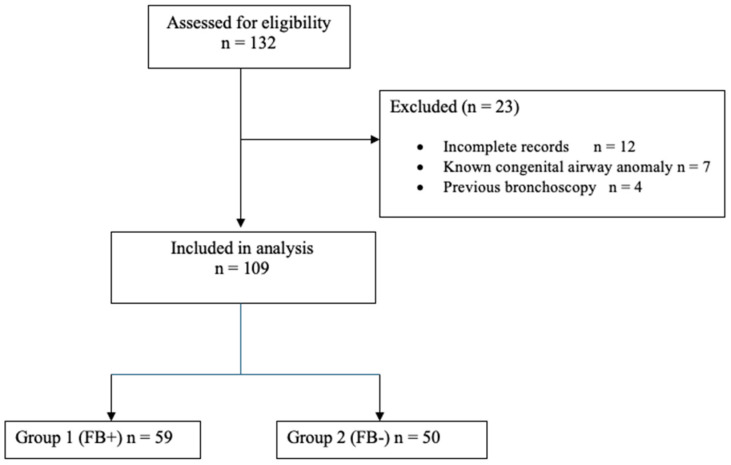
Flow diagram of patient selection.

Between October 2021 and June 2025, patients presenting with suspected FBA were evaluated by thoracic surgeons. A total of 109 patients who continued to be clinically suspected of FBA after history, physical examination, and radiological imaging, and subsequently underwent bronchoscopy, were included in the study. Patients with incomplete records, known congenital airway malformations without suspected foreign body, or prior bronchoscopy during the study period were excluded. The patient selection process is illustrated in Figure 1.

All patients with suspected FBA underwent fiberoptic bronchoscopy (FOB) under general anesthesia in the operating room following insertion of a laryngeal mask airway (LMA). Patients in whom a tracheobronchial foreign body was observed during FOB were classified as Group 1 (n = 59), while those without a foreign body were classified as Group 2 (n = 50). For patients with confirmed FBA, rigid bronchoscopy was performed during the same session to remove the foreign body. Data including age, sex, presence and laterality of foreign body, and preoperative pO_2_, pCO_2_, and SpO_2_ values were recorded. All blood gas values are reported in mmHg, with kPa equivalents provided in parentheses where appropriate (1 mmHg ≈ 0.133 kPa). Additionally, the type of foreign body (organic vs. inorganic) was recorded for Group 1 (confirmed FBA) cases.

Preoperative respiratory and metabolic status was assessed using three complementary modalities:

1. Venous blood gas analysis (Radiometer ABL90 FLEX analyzer, Radiometer Medical ApS, Copenhagen, Denmark) was performed immediately after peripheral intravenous cannulation in the operating room. A 1 mL sample was drawn from the antecubital vein, and pO_2_ (venous) values were recorded in mmHg (with kPa equivalents provided in results).
2. Capnography was performed using a Dräger Infinity Delta monitor with mainstream sidestream sensor (Dräger Medical GmbH, Lübeck, Germany). End-tidal CO_2_ (ETCO_2_) was continuously measured during spontaneous breathing and reported as pCO_2_ (capnography-derived).
3. Pulse oximetry (same Dräger monitor) provided continuous peripheral oxygen saturation (SpO_2_) values, recorded as the average over a stable 3 min period before any sedation or oxygen administration. All measurements were taken after a 5 min stabilization period with the child in a semi-upright position and before induction of anesthesia. The children were breathing spontaneously on room air and were not yet intubated or sedated during the measurement period

Preoperative data were prospectively compared with postoperative results.

Radiological findings (Unilateral hyperinflation, atelectasis and/or pneumonia, and normal chest radiograph) were recorded and compared between children with and without confirmed foreign body aspiration.

### Statistical Analysis 

The distribution of continuous variables was assessed for normality using the Shapiro–Wilk test. Variables with normal distribution were expressed as mean ± standard deviation (SD) and compared between groups using the independent samples t-test. Non-normally distributed variables were reported as median (interquartile range) and compared with the Mann–Whitney U test (none in the present study). Categorical variables were presented as frequencies and percentages and compared using the χ^2^ test or Fisher’s exact test as appropriate. 

95% confidence intervals (95% CI) were calculated for all major between-group differences. To account for age as a potential confounder, a multivariate binary logistic regression model was constructed with confirmed foreign body aspiration as the dependent variable and age, pCO_2_, pO_2_, and SpO_2_ as independent variables. Results were reported as adjusted odds ratios (OR) with 95% CI. 

All statistical analyses were performed using R software version 4.5.1 (R Core Team, Vienna, Austria, 2024). A two-sided *p*-value < 0.05 was considered statistically significant.

## 3. Results

A total of 109 patients were included in the study. Of these, 59 patients (54.1%) in whom a foreign body was detected during bronchoscopy were classified as Group 1, while 50 patients (45.9%) without a detected foreign body were classified as Group 2. The gender distribution was similar between the two groups (Table 1).

**Figure 2:**
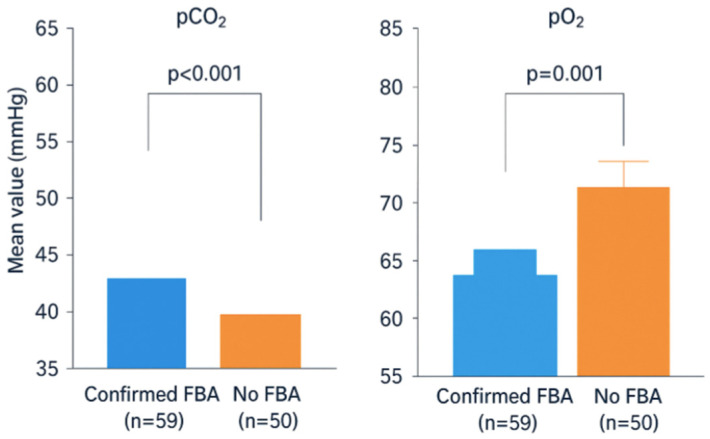
Mean preoperative pCO_2_ (Capnography-derived) and pO_2_ (Venous Blood Gas) levels in children with confirmed foreign body aspiration (FBA) compared with those without FBA. The FBA group showed significantly higher pCO_2_ and lower pO_2_ values, indicating impaired gas exchange due to airway obstruction. Error bars represent the Standard Deviation (SD). pCO_2_: Partial Pressure of Carbon Dioxide (ETCO_2_ value obtained by Capnography device). pO_2_: Partial Pressure of Oxygen (Obtained via Venous Blood Gas (VBG) analysis).

The mean age was 24.63 ± 12.32 months in Group 1 and 37.12 ± 32.98 months in Group 2, with a statistically significant difference between groups (*p* = 0.014). pCO_2_ was significantly higher in Group 1 (41.24 ± 13.37 mmHg) compared to Group 2 (31.53 ± 6.44 mmHg; *p* < 0.001). pO_2_ was lower in Group 1 (45.78 ± 12.18 mmHg) than in Group 2 (53.98 ± 13.24 mmHg; *p* = 0.001) (Figure 2). SpO_2_ was 73.44 ± 17.85 in Group 1 and 77.63 ± 15.64 in Group 2, though this difference did not reach statistical significance (*p* = 0.19).

Multivariate logistic regression adjusting for age confirmed that elevated pCO_2_ (adjusted OR 1.15, 95% CI 1.09–1.22, *p* < 0.001) and reduced pO_2_ (adjusted OR 0.93, 95% CI 0.89–0.97, *p* = 0.001) were independent predictors of confirmed foreign body aspiration, whereas SpO_2_ was not (Table 2).

Among patients with confirmed foreign body aspiration, 33 foreign bodies (56%) were localized in the right bronchial system, 24 (41%) in the left bronchial system, and 2 (3.4%) in the trachea (Table 3). Among the 59 patients in Group 1, 56 foreign bodies (94.9%) were organic (e.g., nuts, seeds), while 3 foreign bodies (5.1%) were inorganic (e.g., plastic fragments, pins). No intraoperative or postoperative morbidity or mortality was observed in any patient.

Distribution of foreign body localization among confirmed cases (Group 1): The right bronchial system was the most frequent site of foreign body impaction (56%), followed by the left bronchial system (41%) and trachea (3.4%).

Additionally, radiological findings differed significantly between the groups (Table 4). Normal chest X-ray was observed in 92% of patients without foreign body, whereas only 45.8% of those with confirmed foreign body had normal imaging (*p* < 0.001). Unilateral hyperinflation was the most common abnormal finding in Group 1 (30.5%).

## 4. Discussion

FBA is a life-threatening condition that requires urgent airway intervention, particularly in children. A detailed history and physical examination remain crucial in suspected cases. The most common clinical findings include cough, choking sensation, wheezing, and unilateral reduction in breath sounds, which should be supported with radiological assessment. However, normal imaging does not exclude the diagnosis of FBA.

In this prospective study, patients in Group 1 had a significantly lower mean age (24.63 ± 12.32 months) compared to Group 2 (37.12 ± 32.98 months; *p* = 0.014). This finding is consistent with previous pediatric FBA studies that report a high-risk profile in children under the age of three [8,9,10]. Large multicenter studies have shown that 50–80% of cases occur in the 1–3-year age group, while aspiration is less frequent but associated with higher morbidity and mortality in infants under one year of age [11]. In flexible bronchoscopy series, the median age is reported around 24 months, reflecting that most cases involve toddlers who have just begun walking and chewing [12]. Developmental factors, such as immature neuromotor coordination, lack of posterior teeth, oral exploratory behavior, and concurrent activities during feeding, increase the risk of FBA in younger children; therefore, both literature data and our findings suggest that the diagnostic threshold for suspicion should be lower in children under three years of age [13].

Capnography, or continuous end-tidal carbon dioxide monitoring, is a non-invasive method that provides real-time assessment of CO_2_ levels in the body. CO_2_, produced during cellular metabolism, is transported to the lungs via circulation and exhaled. Continuous end-tidal CO_2_ monitoring provides valuable information about ventilation, metabolism, and circulation. It is commonly used in emergency departments, operating rooms, intensive care units, and during patient transport, particularly to confirm endotracheal tube placement [14]. During cardiopulmonary resuscitation, changes in cardiac output are reflected in end-tidal CO_2_ concentrations, and during sedation, capnography has been shown to detect respiratory failure earlier than pulse oximetry or respiratory rate alone. In our study, pCO_2_ levels were significantly higher in Group 1 (41.24 ± 13.37 mmHg), while pO_2_ levels were significantly lower (45.78 ± 12.18 mmHg). These findings demonstrate that FBA causes mechanical airway obstruction, restricting alveolar ventilation and resulting in alveolar hypoventilation with subsequent CO2 retention (hypercapnia), and impaired ventilation-perfusion balance, leading to hypoxemia [8,9,15,16]. Respiratory acidosis may become particularly severe in cases of delayed diagnosis or bilateral obstruction [17].

In partial obstructions, a “ball-valve” mechanism may occur due to airflow imbalance, leading to air trapping and further impairment of CO_2_ elimination [15]. The degree of hypercapnia correlates with the level and severity of obstruction, being more pronounced in proximal tracheobronchial occlusions [11]. In children, narrower airway anatomy predisposes to earlier ventilation-perfusion mismatch, resulting in a rapid rise in arterial CO_2_ pressure [18]. Recent clinical studies have also demonstrated that elevated pre-bronchoscopy pCO_2_ is an important predictor of foreign body presence [12,19].

Airway obstruction disrupts alveolar ventilation, leading to hypoxemia, which is most commonly manifested by reduced SpO_2_ levels [7]. However, in our study, no significant difference in oxygen saturation was observed between Group 1 (73.44 ± 17.85) and Group 2 (77.63 ± 15.64) (*p* = 0.19). The mean preoperative SpO2 values (73–78%) may appear unusually low for clinically stable children; however, this cohort consisted of highly symptomatic patients evaluated urgently. The measurements were obtained in the operating room environment on room air, before any sedation or oxygen supplementation. Many toddlers were anxious or crying intermittently, a well-recognized trigger for transient desaturation in partial airway obstruction [20].

Several possible explanations for the lack of significant difference between the groups are supported by the literature. First, in partial obstructions, airway patency may be partially preserved, allowing baseline SpO_2_ values to remain within normal limits, especially at rest [21]. Moreover, children possess strong compensatory mechanisms, and SpO_2_ may remain stable until hypoxemia develops, thereby masking early desaturation [10,15,22]. Indeed, some studies have demonstrated that the location and size of the foreign body are more predictive of clinical manifestations, while SpO_2_ tends to decline significantly only in cases of complete obstruction or severe ventilation–perfusion mismatch [20,21,22]. Finally, other studies have reported that low SpO_2_ levels are more commonly associated with delayed diagnoses or complicated cases, while no significant differences are seen in patients who present early [19].

Therefore, the lack of a significant association between SpO_2_ and FBA in our study appears consistent with the pathophysiological and clinical factors described in the literature.

Regarding localization, foreign bodies were detected in the right bronchial system in 56% of cases, the left bronchial system in 41%, and the trachea in 3.4%. This distribution aligns with previous studies, as the anatomical structure of the right main bronchus predisposes to higher rates of foreign body lodgment [15,16,18]. Although less frequent, tracheal foreign bodies may result in more severe respiratory compromise [23].

Regarding the characteristics of the aspirated materials, the overwhelming majority of foreign bodies found in Group 1 were organic (94.9%), aligning with previous large pediatric series which commonly cite nuts and seeds as the predominant aspirated materials. This high prevalence of organic material is crucial for interpreting our gas exchange findings. Organic foreign bodies often cause more severe airway mucosal inflammation, swelling, and greater bronchial obstruction due to their hygroscopic nature, potentially leading to a more pronounced ventilation-perfusion mismatch and, consequently, the significantly higher pCO_2_ and lower pO_2_ levels observed in our FBA cohort. While other variables such as size and chronicity were not analyzed, the strong predominance of organic materials in our study supports the finding of severe physiological compromise, which was the focus of our investigation.

Bronchoscopy remains the gold standard for both the diagnosis and treatment of FBA [23]. However, to avoid unnecessary procedures, integration of radiological findings, clinical history, and gas exchange parameters is recommended in selected cases [24]. Furthermore, developing algorithmic approaches based on gas exchange parameters and clinical profiles prior to bronchoscopy may play an important role in minimizing invasive interventions [10].

In cases where no foreign body was detected (Group 2), symptoms and findings were likely attributable to other respiratory pathologies such as viral infections, asthma, or bronchitis. Combining clinical and radiological findings increases diagnostic accuracy [8,9,10,15], which may partly explain why bronchoscopy was performed in some of these patients. The presence of these underlying inflammatory or obstructive conditions, which can also affect gas exchange, is implicitly acknowledged as a limitation of our study. While Group 2 exhibited significantly lower pCO_2_ and higher pO_2_ levels compared to Group 1, reflecting generally better gas exchange, it is plausible that the observed mean values for Group 2 are slightly altered by these mimics. Nevertheless, the clear distinction in gas exchange parameters supports the utility of blood gas analysis in distinguishing the more profound physiological impact of FBA from that of common pediatric respiratory mimics

This study has certain limitations. Being single-center reduces the generalizability of the findings. Additionally, while we included the type of foreign body, variables such as size and duration of aspiration and onset of symptoms were not included in the analysis. Future multicenter studies with larger cohorts should consider these parameters to provide a more comprehensive evaluation [9,16,18].

Table 5 presents the main differential diagnoses that should be considered when similar blood gas abnormalities (elevated pCO_2_ and/or reduced pO_2_) are observed in children with suspected foreign body aspiration. A witnessed choking episode and unilateral radiological findings remain the most reliable discriminators for FBA.

## 5. Conclusions

In our study, patients with bronchoscopy-confirmed foreign body aspiration were found to be younger in age, with elevated pCO_2_ and reduced pO_2_ levels. Based on these results and the current literature, we suggest that in patients aged 1–3 years presenting with suspected foreign body aspiration, pCO_2_ values above the normal range (35–45 mmHg) and pO_2_ values below normal may strongly support the likelihood of a tracheobronchial foreign body. However, given the high variability in pCO2 (SD ±13.37 mmHg) and potential clinical overlap, these parameters should be interpreted cautiously and strictly within the context of the overall clinical picture.

Although the difference in SpO_2_ did not reach clinical significance, our findings indicate that gas exchange parameters may serve as useful adjuncts in the diagnostic process. Furthermore, the predominance of the right bronchial system as the most common site of foreign body lodgment was reaffirmed. Clinical decision-makers should consider age, blood gas parameters, and the likelihood of localization collectively prior to bronchoscopy, in order to avoid unnecessary invasive procedures and enhance patient safety.

## Figures and Tables

**Table 1:** Comparison of demographic, venous blood gas, and non-invasive parameters between groups.

Parameter	Group 1 (n = 59, with FB)	Group 2 (n = 50, Without FB)	*p*-Value
**Age (months)**	24.63 ± 12.32	37.12 ± 32.98	0.014 *
**Sex (male/female)**	37/22	33/17	>0.05
**pCO_2_ (mmHg) ^‡^**	41.24 ± 13.37 (5.49 ± 1.78 kPa)	31.53 ± 6.44 (4.20 ± 0.86 kPa)	<0.001 ***
**pO_2_ (mmHg) ^§^**	45.78 ± 12.18 (6.10 ± 1.62 kPa)	53.98 ± 13.24 (7.18 ± 1.76 kPa)	0.001 **
**SpO_2_ (%) ^‖^**	73.44 ± 17.85	77.63 ± 15.64	0.19 (ns)

FB = Foreign body; ns = not significant. * *p* < 0.05, ** *p* < 0.01, *** *p* < 0.001. ^‡^ pCO_2_: Partial Pressure of Carbon Dioxide (ETCO_2_ value obtained by Capnography device). ^§^ pO_2_: Partial Pressure of Oxygen (Obtained via Venous Blood Gas (VBG) analysis). ^‖^ SpO_2_: Peripheral Oxygen Saturation (Obtained via Pulse Oximetry).

**Table 2:** Multivariate logistic regression analysis for predictors of confirmed foreign body aspiration (adjusted for age).

Variable	Adjusted Odds Ratio	95% Confidence Interval	*p*-Value
**Age (months)**	0.96	0.93–0.99	0.018
**pCO_2_ (mmHg)**	1.15	1.09–1.22	<0.001
**pO_2_ (mmHg)**	0.93	0.89–0.97	0.001
**SpO_2_ (%)**	0.98	0.95–1.02	0.321

**Table 3:** Distribution of foreign body localization (Group 1).

Localization	n	Percentage (%)
Right bronchial system	33	56.0%
Left bronchial system	24	41.0%
Trachea	2	3.4%

**Table 4:** Radiological findings in patients with and without confirmed foreign body aspiration.

Radiological Finding	Group 1 (with FB, n = 59)	Group 2 (Without FB, n = 50)	*p*-Value *
Normal	27(45.8%)	46(92.0%)	<0.001
Unilateral hyperinflation	18(30.5%)	2(4.0%)	<0.001
Atelectasis and/or pneumonia	10(16.9%)	2(4.0%)	0.046
Hyperinflation + atelectasis/pneumonia	4(6.8%)	0(0%)	0.125
**Total**	**59(100%)**	**50(100%)**	-

* *p*-values were calculated using the χ^2^ test or Fisher’s exact test as appropriate. FB = foreign body.

**Table 5:** Differential diagnosis of abnormal blood gas parameters (↑ pCO_2_ and/or ↓ pO_2_) in children with suspected foreign body aspiration.

Condition	Typical Age Group	Key Clinical Features	Blood Gas Pattern	Imaging Findings	Discriminator for FBA
Foreign body aspiration (FBA)	1–3 years	Sudden onset, witnessed choking episode	↑ pCO_2_, ↓ pO_2_, normal–low SpO_2_	Unilateral hyperinflation, atelectasis	Witnessed event + unilateral findings
Acute asthma exacerbation	Any age	Recurrent wheezing, family history of atopy	↓ pO_2_, normal– ↓ pCO_2_	Hyperinflation (bilateral)	Response to bronchodilators
Viral bronchiolitis/pneumonia	<2 years	Fever, rhinorrhea, gradual onset	↑ pCO_2_, ↓ pO	Bilateral perihilar infiltrates	Viral prodrome, fever
Bacterial pneumonia	Any age	High fever, focal chest signs	↑ pCO_2_, ↓ pO_2_	Lobar consolidation	Fever + leukocytosis
Croup (laryngotracheobronchitis)	6 months–3 years	Barking cough, stridor, hoarseness	Mild ↑ pCO_2_	Steeple sign	Stridor + inspiratory distress
Congenital airway malformation	Neonatal–infant	Chronic symptoms since birth	Variable	Cystic lesions, hyperinflation	Present from birth
Acute epiglottitis	2–7 years	Drooling, tripod position, high fever	Severe ↑ pCO_2_, ↓ pO_2_	Thumb sign on lateral neck X-ray	Rapid progression + drooling
Cardiac failure (left-to-right shunt)	Infant–child	Tachypnoea, hepatomegaly, failure to thrive	↓ pO_2_, normal–mild ↑ pCO_2_	Cardiomegaly, pulmonary venous congestion	Cyanosis + cardiomegaly

## Data Availability

The research data can be requested from the corresponding author. The data are not publicly available, as the authors want to carry out further analyses and publish them before the data can be made available to the public.
